# Description of Extrudate Swell for Polymer Nanocomposites

**DOI:** 10.3390/ma3010386

**Published:** 2010-01-12

**Authors:** Kejian Wang

**Affiliations:** Institute of Plastics Machinery and Engineering, Beijing University of Chemical and Technology, Beijing 100029, China; E-Mail: wangkj@mail.buct.edu.cn; Tel.: +86-10-64435015; Fax: +86-10-64435015

**Keywords:** extrudate swell, nanocomposite, capillary, concentration shift factor

## Abstract

Extrudate swell is often observed to be weakened in nanocomposites compared to the pure polymer matrix. A theory quantifying this would be significant either for optimum processing or for understanding their viscoelasticity. A unified extrudate swell correlation with material properties and capillary parameters was suggested for polymer melt and their nanocomposites when considering the reservoir entry effect. More importantly, it was the first to find that the composite swell ratio can be the matrix swell ratio multiplied by the concentration shift factor, which is similar to the dynamic moduli expression for composites. The factor is a function of the shear field (stress or shear rate), filler content, filler internal structure and the surface state as well as the matrix properties. Several sets of swell data for nanocomposites were chosen from publications to test the new theories. The proposed quantitative model displayed good fit for the five kinds of nanocomposites, which verified the rationality of the swell theory for nanocomposites.

## 1. Introduction

In general, incorporation of reinforcing filler into polymers may result in substantial improvement of mechanical, dynamic and thermal properties of the polymer matrix. Polymeric nanocomposites are generally regarded as a potential substitute for conventional microcomposites and the need for a better understanding of filler reinforcement has led to investigations into the mechanisms. The melt processing of nanocomposites requires information about their rheological properties, which are sensitive to many factors, such as the processing conditions, the polymer– filler interaction and the structure and morphology of the system. Up to now, some general conclusions have been drawn concerning the rheological behavior caused by the filler particles. Some have been well documented. For instance, entirely new properties such as yield behavior, high hysteresis, stress softening (Mullins effect), and strain dependent dynamic modulus (Payne effect) and thixotropy are regarded as special characteristics when compared to the unfilled polymer; others concern the enhanced nonlinear behaviors or reduced behaviors, e.g., post-extrusion swell. The die swell, also called extrudate swell or Barus effect is an important phenomenon determining the shape/size and quality of extrudate products. Better understanding will provide useful guidelines for optimizing processing conditions, the design of polymer processing equipment, the ability to predict the energy requirements and correlations with the structural evolution of composites [10]. Moreover, the extrudate swell can be used to assess the elasticity of the polymer upon melt extrusion. When a molten polymer flows through a capillary, molecular chains become oriented, uncoiled or disentangled due to the applied shear. The entanglements will, to some extent, prevent the molecules from slipping past one another, thus preventing total relaxation of the molecules. As the melt leaves the die, molecular chains tend to recoil in the flow direction and grow in the normal direction, leading to extrudate swell. The mechanism and degree of swelling are usually explained in terms of elastic recovery or effect of residence time on the applied stresses. Regardless of the extensive experimental results, to our knowledge, there are few quantitative theories for describing the swell of filled composites, which is analyzed in the present study.

The mechanisms for reinforcement and nonlinearity of the filled polymers still remain controversial. A widely held view is that filler aggregation-agglomeration and three dimensional network formation are responsible for the high levels of reinforcement and that deagglomeration and network breakdown are responsible for the nonlinearity with strain. It is generally believed that reinforcement comes from four different sources: the pure polymer network, the hydrodynamic effect introduced by filler particles, specific filler–polymer interactions, and a strain-dependent part caused by the filler network. Filler network is formed even by Van der Waals interactions, electrostatic bonds, or possibly confinement. Thus, there are many dynamic models considering filler agglomeration–deagglomeration of a percolating filler network, matrix-filler bonding and debonding or adhesion and de-adhesion of polymer chains with varying chain mobility at the filler interface The complex interactions modify chain dynamics due to the disentanglement of matrix polymer chains from those bound to the filler particle. The bridging of particles by long chains appears to broaden the interaction range of the filler structure. Filler-filler interactions, which are dependent on the particle size, shape, surface area, the distribution, dispersion and concentration of the inclusions in the matrix, become significant in the molecular structure of the polymer [11,12]. The present focus is not the microscopic mechanism but rather a quantitative description of die swell reflecting the variation in structure.

Structure variations bring about enhanced nonlinear viscoelastic responses [13]. Additionally, the extrudate swell in the capillary is related to the melt elasticity and the applied shear rate/stress, temperature, L/D ratio, and the presence of fillers. Sadhu *et al.* [14] reported that the extrudate swell decreased with increasing clay content in three types of rubber based nanocomposite (styrene butadiene rubber, acrylonitrile butadiene rubber and polybutadiene rubber). Modified-clay nanocomposites exhibited a lower die swell than unmodified nanocomposites, which was attributed to better dispersion/exfoliation of modified-clay in the rubber matrix. Most importantly, here will show that the extrudate swell of the composite can be expressed as that of the corresponding matrix polymer multiplied by a coefficient which is similar to the concentration shift factor used in describing the dynamic modulus variation with the filler loading level [15].

Based on the above structural origins, the present theory for composites will be obtained from the quantitative theories of the extrudate swell for the pure polymeric melts [16,17]. There are some correlations relating the swell ratio to rheological parameters [18]. In the 1970s, Bagley and Duffey [19], Graessley *et al*. [20], Han [21] and Tanner [22] proposed expressions describing relationships between the swell ratio B and the first normal stress difference or shear stress on the supposition that polymer melt shear flow obeyed simple laws. The most famous systematic theory of extrudate swell of entangled polymeric liquids is that of Tanner [23], who related the maximum diameter of the extrudate to the recoverable shear strain (S_R_) at the capillary wall on the basis of K-BKZ constitutive equation in Poiseuille flow [17]. The relationship was later re-confirmed for a wide class of constitutive equations, including PTT, Pom–Pom and general network type models for fully-developed tube flow [23]. Regardless of the fact that the swell could be related well with elasticity of polymeric fluids, the viscous heating and the time-dependent nature of swell are not all deliberated. Considering such two factors, Song [24] established a primary theoretical framework for relating the extrudate swell to the intrinsic viscoelasticity and exterior conditions. The extension and flow can also induce the dynamic and reversible disentanglement and reentanglement between polymeric chains such that the polymeric melts then undergo a partial stress relaxation and produce extrudate swell ratio at the die exit. Based on the O-W-F constitutive equation and the multiple transient-network model as well as the double relaxation dynamics of reentanglement-disentanglement transition (RE-DT) and recoil-uncoil transition (RC-UCT) from the Poiseuille flow [25,26]. A new set of swell equations as functions of molecular parameters, operational parameters and growth time under the steady state and dynamic state were developed. Song’s model successfully described the die swell through a long capillary of linear polyethylene (HDPE) and linear polybutadiene (PBD) with the different molecular weights for different processing variables [27].

However, Both Tanner and Song assumed that the chain elongation incurred at entry is fully relaxed. This is almost true for extrusion in long capillaries, but for short capillaries, the entry effect figures prominently and entrance effects are relatively numerous and complicated in short-tube flow of polymer melts [1,3,9,11,28,29]. For the case of a short capillary, Liang [11] obtained some semi-empirical quantitative relationships of the swell ratio with material characteristics and operational parameters [30]. Thus, it is urgent to modify Song’s model to describe the swelling behaviors for extrusion in short capillaries for either pure polymers or their composites. The paper is organized as follows. Section 2 develops the quantitative model. In Section 3 some swell experimental data were used to verify the developed theory. Section 4 deals with concluding remarks.

## 2. Theoretical Analysis

### 2.1. Extrudate Swell Theory for Long Capillary Extrusion

In capillary extrusion, polymer flows from a reservoir through a capillary to swell out of the exit. The molecular chains are oriented in the convergent region resulting in an entry effect while the swelling evolves with time to be maximum outside of the capillary. Therefore, the effects of entry, capillary flow field and outside environment conditions should all be considered in establishing a systematic swell theory.

Different from the previous die swell expressions incorporating the power law model, one viscosity model in the steady shear flow was derived from O-W-F constitutive equation together with the molecular dynamics by Song [25]:

(1) $$\eta (\dot{\gamma })=\eta_{0}/{\left[1+{\left(\tau_{0}\dot{\gamma }\right)}^{a}\right]}^{\text{n}}$$

It will later be shown that Equation 1 is successful for pure polymers and most particle-filled composites. From experimental data of η, the molecular parameters of typical relaxation time $\tau_{0}$, field-dependent exponents n and a can be determined. $\eta_{0}$ is viscosity at $\dot{\gamma }=0$. When assuming the elongated chain at the entry region from the reservoir was fully relaxed, the ultimate extrudate swell *B* is approximately obtained as in Equation 2 by Song [27]:

(2) $$B=\frac{1}{2}{\left({\left(n{\left(\frac{\eta_{0}}{G_{0}}\right)}^{1/n}\dot{\gamma }/(1+{\left(L/D\right)}^{\alpha })\right)}^{\left(1-W\right)}+5.098\right)}^{1/2}$$

*B* depends on molecular parameters and the operational variables [L/D and $\dot{\gamma }$ or τ]. (1−W) is the fraction of the recoverable conformation of the disentangled polymeric chain in the flow. For the details of the above equations (1−2) and the applications for HDPE and PDB please refer to [27].

### 2.2. Extrudate Swell Theory for Short Capillary Extrusion

In a short capillary, the entry flow is more complicated. Liang [31] presented the die swell ratio as follows:

(3) $$B={\left(1+\lambda_{l}S_{R}\right)}^{1/2}$$

(4) $$S_{R}={\left(N_{1}/2\tau \right)}^{1/2}$$

where $S_{R}$ is the recoverable shear strain as defined by Equation 4. The coefficient $\lambda_{l}$ shows the elastic strain by the stored energy in the capillary reservoir, which is related to the capillary structure and the fluid viscoelasticity. The factor $\lambda_{l}$ is a function of the entry converging flow parameter. The recoverable strain can also be estimated from the experimental swell ratio by Tanner’s Equation (5) [17]:

(5) $$S_{R}={\left[2(B^{6}-1)-2\right]}^{0.5}$$

Equation 3 is similar to Song’s approximation in equation (2) of the total die swell ratio, which may be revised to describe the extrudate swell from the short capillary as in Equation 6:

(6) $$B={\left(k_{t}k_{L\dot{\gamma }}\frac{M}{4}{\left(S_{R}\right)}^{\left(1-W\right)}+5.098/4\right)}^{f_{w}}$$

where $k_{L\dot{\gamma }}={\left[\frac{1+{\left(\tau_{0}\dot{\gamma }\right)}^{a}}{1+{\left(L/D\right)}^{a}}\right]}^{\left(1-w\right)}$ shows the effects of capillary length, in which molecular relaxations occur. M/4 is the recoverable effect from the stored energy in capillary reservoir. It may be *ca.*1 for a adequately long capillary, while it may be $\lambda_{l}$ for a very short capillary as in Equation (3). $k_{t}$ was multiplied to consider the extrudate swell evolution out of the capillary, in which molecular dynamics also exhibits disentanglement-reentanglement transitions and uncoil-recoil transitions as in a capillary [27]. $k_{t}$ is almost one when the maximum swell was approached at $t=t_{\infty }=\infty$ as in Song’s work [27]. Here $f_{w}$ is one component compensating for the approximation from Song’s original derivation [27]. Thus, Equation (6) may be useful for both long and short capillaries for including many relative factors regardless of the treatment of such phenomena. For simplicity in application, Equation (6) can also be rewritten as Equation (7):

(7) $$\text{B}={(\frac{k_{\text{tlM}}}{4}{\text{*(n*}\tau_{0})}^{\text{(1/n)}}\dot{\gamma }\text{/(1}+{(L/D\text{)}}^{\text{a}}\text{))}}^{\text{(1-w)}}+{\text{5.098/4)}}^{\mathrm{fw}}$$

where $k_{tlM}=k_{t}k_{L\dot{\gamma }}M$.

### 2.3. Addition Effects of Filler in Extrudate Swell of Filled Composites

In the particle-filled composites, filler is distributed in the entangled matrix network. The filling affects the network relaxation [31]. Regardless of the variation in viscoelastic properties, experiments have found that the η of filled composite are similar to those of pure polymer matrix. *i.e.*, the above extrudate swell theory may be modified for the filled composites.

However, experiments showed that the die swell was usually weakened with the increment of filler concentration [32]. The observation for the experimental data of composite die swell inspires the following correlation of Equation (8):

(8) $$B(\dot{\gamma },\phi )=B(\dot{\gamma },\phi =0)f(\phi )$$

Here $B(\dot{\gamma },\phi )$ and $B(\dot{\gamma },\phi =0)=B_{m}$ are the die swell of the composite with the filler content of ϕ and the die swell of the pure matrix respectively. f(ϕ) represents the filling effects. Equation (8) indicates that the viscoelastic behavior of the filled composites is dominated by the elasticity of the composite matrix in the high shear rate range. Liang [32] found that $B=\lambda \tau_{w}(1-\phi \varsigma )$, where $\tau_{w}$ is wall shear stress, λ and ς are constants related to the elasticity of matrix melt and the geometry of the filler particles. The function f(ϕ) is analogous to those used for the viscosity and modulus of a suspension of spheres, and may be called “concentration shift factor” [33]. There are several forms for f(ϕ). Here it is written as:

(9) $$f(\phi )=1-{\left(\phi /p_{c}\right)}^{q}$$

where $p_{c}$ is the equivalent percentage illustrating the decrement of the network elasticity by filling the particles, which is directly related to the shear rate $\dot{\gamma }$ as described probably by $p_{c}={\left(\dot{\gamma }/{\dot{\gamma }}_{c}\right)}^{p}$ or to shear stress by $p_{c}={\left(\tau /\tau_{c}\right)}^{p\prime }$. ${\dot{\gamma }}_{c}$ and $\tau_{c}$ is the limit shear rate and the limit shear stress respectively when the network is almost completely destroyed. It seems that $p_{c}=x\phi_{c}$, where $\phi_{c}$ is the critical volume percentage in the percolation theory. Experimental observations also show that:

(10) f(ϕ)=b(ϕ)−l(ϕ)τ

This expression focuses on the effects of shear stress on the concentration shift factor. The factors q, b and l are constants depending on the filler-polymer system and the flow field.

## 3. Verification of the Theory of Die Swell for Nanocomposites

### 3.1. Experimental

To verify the above swell theory in nanocomposites, experimental die swell data for organo-bentonite-filled polypropylene nanocomposites [34], calcium carbonate nanoparticle-filled isotactic polypropylene [35], clay filled fluoroelastomer nanocomposites [36], quasi-nanogel particles filled natural rubber [29] and electron beam-cross-linked gels filled natural rubber [37] are chosen The experimental details can be found in the corresponding publications. The different key points with regards to rheometry are summarized as follows.

For organobentonite-filled polypropylene nanocomposites [34], the used resin was polypropylene (Moplen HP550R, HMC Polymers Co. Ltd., Thailand) with MFI = 22 g/min. Polypropylene-grafted maleic anhydride (PP-g-MA, grafted MA at 1 wt %l) was the compatibilizer at the weight percent of 15%. Na-Bentonite (Mac-Gel, Grade SAC) was modified with hexadecyltrimethylammonium [C_16_H_33_N+(CH3)_3_] bromide salt before being mixed into resin at different levels. Melt rheology and extrudate swell studies were carried out by using a CEAST Rheologic 500 twin bore capillary rheometer. The inner diameter and the length of the barrel used were 15 and 300 mm, respectively, while the length-to-inner diameter (L/D) of the circular die was 20/1.

For PP/nano-Ca_2_CO_3_ [35], the resin was copolymer iPP (Moplen CS-42 HEXP, HMC Polymers Co., Ltd Thailand) with MFR (2.16 kg at 230 °C) of 12 g/(10 min). The filler were cubic CaCO_3_ nanoparticles NPCC-111 and NPCC-201 with average particle size of 40 nm and purity ≥97%. The surface of NPCC-201 grade was partially modified with stearic acid to facilitate particle dispersion and distribution within the matrix, while that of NPCC-111 grade was uncoated. A CEAST Rheologic 5000 twin-bore capillary rheometer was used to measure shear viscosity and extrudate swell. The inner diameter and the length of the barrel used were 9.95 and 250 mm respectively, while the inner diameter and the length of the die were 2 and 15 mm (*i.e.,* L/D ratio = 7.5), respectively. The testing temperature was calibrated at 190 ± 0.5 °C.

For clay filled fluoroelastomer nanocomposites [36], fluoroelastomer (FKM, VITON A-500) from DuPont Dow elastomers was used as matrix, The filler was O-MMT (Organo-MMT, quaternary ammonium salt of dimethyl hydrogenated tallow: Cloisite 15A from Southern Clay Products, Inc., USA). The flow properties and extrudate swell ratio of pristine and filled FKM were measured by a Monsanto processability tester (MPT).The capillary used in our investigation had a length to diameter (L/D:22.9/0.762 mm) ratio of 30:1 with compound entrance angles of 45° or 60°.

For quasi-nanogel particles filled natural rubber [29], natural rubber (NR) latex was vulcanized by sulfur in the presence of zinc diethyl dithiocarbamate (ZDC) as an accelerator and zinc oxide (ZnO) as an accelerator activator. The sulfur-to-accelerator ratios were kept at 3, 2, 1, 0.5, and 0.33 leading to the final gel percentages of 97, 96, 94, 88, 86 and crosslingk density of 0.27, 0.36, 0.75, 10.0, 1.11 respectively. The melt flow properties of the NR samples with and without gel were measured with a Monsanto processability tester (Monsanto Company, Akron, Ohio) (barrel radius 59.53 mm). The capillary had a length-to-diameter ratio equal to 30 (length = 30.0 mm, diameter = 1.0 mm).

For electron beam-cross-linked gels-filled natural rubber [37], the latex samples were subjected to electric beam (EB) radiation at various irradiation doses. In radiation, some samples were sensitized by butyl acrylate. NR latex gel were then mixed into raw NR forming the filled natural rubber. The rheological tests were similar to those in reference [29].

### 3.2. Comparison of the Predicted Values with the Experimental Data

#### 3.2.1. Application of the Swell Model to PP/Organobentonite Composite

**Figure 1:**
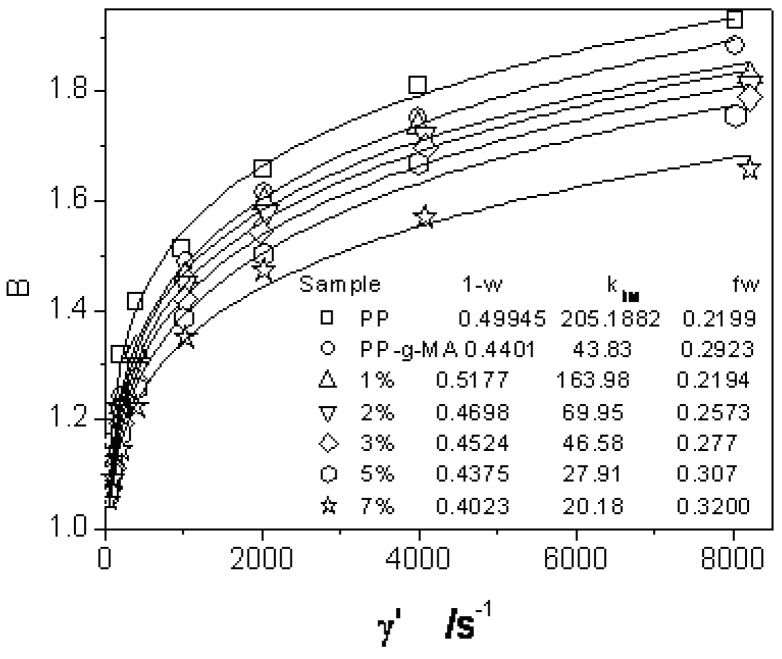
The die swell ratio of iPP/organobentonite nanoparticles of different filler contents.

The constitutive parameter values of iPP and iPP/organobentonite were obtained by fitting the viscosity curves [34] as listed in Table 1. For low loading of inorganic filler, it is found that n and a remain almost constant for different filler concentrations. However, the zero shear viscosity becomes larger with the longer apparent relaxation time for higher organobentonite-loaded composites. This regular variation was due to the fact that maleic anhydride (PP-g-MA) compatibilized polypropylene with bentonite which was also modified with bromide salt before being mixed into resin. *i.e.*, filler was mixed into the entangled polymer chain network, which prevented the chain relaxtions and thereafter macroscopic rheological behaviors. The extrudate swell data are well fitted by Equation (7) when L/D = 20/1, as shown in Figure 1. It is noticed that (1−W) and $k_{tlM}$ decrease while $f_{w}$ increases when PP was loaded with more organobentonite. (1−W) gradually deviates from 0.4995 for PP (almost 0.5 as predicted by Song [27]), which illustrates that the percentage of recoverable PP chain conformation after capillary exit reduced, which is consistent with the delayed relaxation in more filler concentrated PP. Simultaneously, smaller $k_{tlM}$ shows the entry effect was weakened for ‘rigidized’ melt.

**Table 1:** The constitutive parameter values of iPP and iPP/Ca_2_CO_3_.

**Φ**	*n*	*τ*_0_ /**s**	*a*	*η*_0_ /**Pa.s**
PP	0.835	0.021	0.84	780
PP-PP-g-MA		0.0213		810
1%		0.0217		850
2%		0.02173		890
3%		0.02237		920
5%		0.02253		970
7%		0.0268		1170

**Figure 2:**
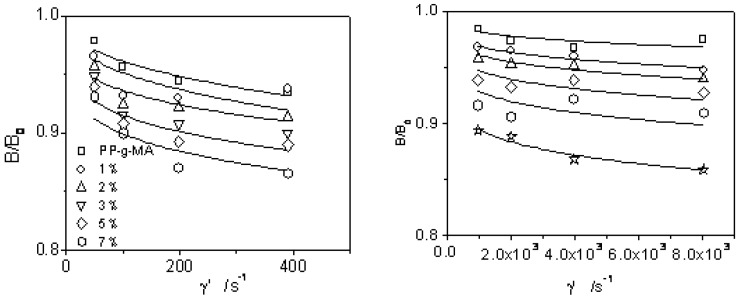
$B(\dot{\gamma },\phi )/B(\dot{\gamma },\phi =0)$ *versus* shear rate for PP/organobentonite nanocomposites.

$B(\dot{\gamma },\phi )/B(\dot{\gamma },\phi =0)$ is calculated against shear rate as in Figure 2 to obtain f(ϕ) as listed in Table 2. It is noted that there is one $\text{f}(\phi )=1-\phi /p_{c}$ for each filler loading level when $p_{c}={\left(\dot{\gamma }/{\dot{\gamma }}_{c}\right)}^{p}$. Therefore, Equation (8–9) indeed fit for the nanocomposite.

**Table 2:** The parameter values in $p_{c}={\left(\dot{\gamma }/{\dot{\gamma }}_{c}\right)}^{p}$ for PP/organobentonite nanocomposites.

**Samples**		f(ϕ) **at low shear rate**		f(ϕ) **at high shear rate**	
		${\dot{\gamma }}_{c}$	p	${\dot{\gamma }}_{c}$	p
PP-g-MA		2.5E6	0.4153	3.0E9	0.2680
Composites with different bentonite content	1%	3.7E5	0.3702	4.0E9	0.2278
	2%	5.4E6	0.2523	5.0E9	0.2100
	3%	7.0E6	0.2356	6.0E9	0.1903
	5%	8.0E6	0.2183	7.0E9	0.1673
	7%	1.0E7	0.1993	8.0E9	0.1415

$p_{c}$ is the exponential function of shear rate. The percolated shear rate ${\dot{\gamma }}_{c}$ increases while p decreases with bentonite content. For the larger aggregates of well dispersed compatible nanoparticles in matrix, the shear rate to breakdown is larger for higher loaded PP. More importantly, $p_{c}$ exhibits different percolation behaviors at low shear rate and at high shear rate as shown in in Figure 2 and Table 2. At the same filler level, ${\dot{\gamma }}_{c}$ greatly increases while p decreases with increasing shear rate. However, $B(\dot{\gamma },\phi )/B(\dot{\gamma },\phi =0)$ increases at medium shear rate (not shown here), which represents one percolation transition stage. In other words, the filler-polymer network and pure polymer network were deformed in shear probably in different mechanisms.

#### 3.2.2. Application of Swell Model to PP/Ca_2_CO_3_ Composite

**Figure 3:**
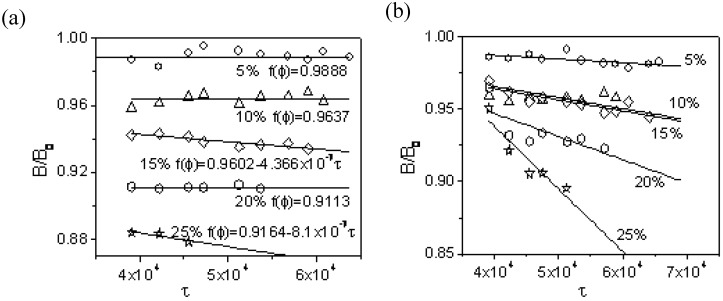
The die swell of iPP/stearic acid-coated (a) and uncoated (b) CaCO_3_ nanoparticles of different filler loadings.

Many experimental results have reported that extrudate swell rose linearly with shear stress [35]. It is noticed that B(τ,ϕ)/B(τ,ϕ=0) reduces almost linearly as shown in Figure 3 for PP/Ca_2_CO_3_ nanocomposite. B(τ,ϕ)/B(τ,ϕ=0) is lower than 1 and drops with filler concentration. Linear function of f(ϕ) *versus* stress as Equation 10 is also confirmed in Table 3.

**Figure 4:**
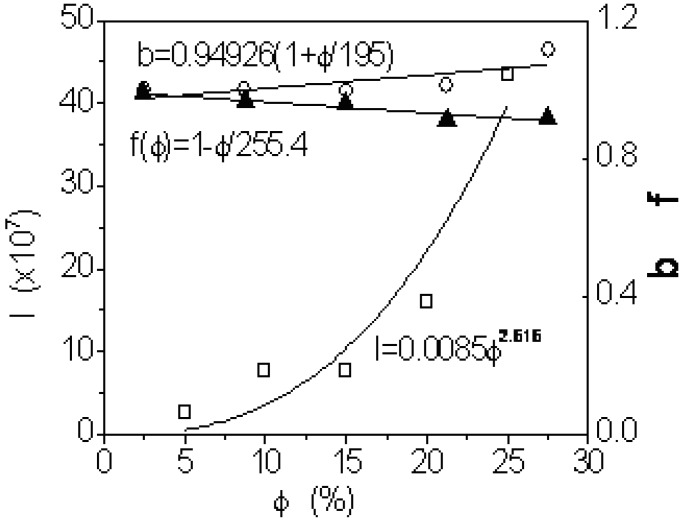
f(ϕ) for coated CaCO_3_/ iPP and b,1 for iPP/uncoated Ca_2_CO_3_.

After surface-coated, Ca_2_CO_3_ particles were well dispersed in PP entangled network forming a double-phase mesoscopic dimensional structure, which is relatively stable. This may explains why the slope of B(τ,ϕ)/B(τ,ϕ=0) *versus* stress is smaller for PP/CaCO_3_ partially modified with stearic acid than for PP/Ca_2_CO_3_ unmodified at the same filler concentration. Most curves are horizontal regardless of the quantity of stress for PP/ modified Ca_2_CO_3_. However, the decrement tendency becomes stronger for higher unmodified Ca_2_CO_3_ in PP. At high shear stress, B(τ,ϕ)/B(τ,ϕ=0) is even lower for unmodified Ca_2_CO_3_/PP than for modified Ca_2_CO_3_/PP. This is due to the structural difference. Strongly excluded unmodified Ca_2_CO_3_ was more easily squeezed out. Thus, the unmodified Ca_2_CO_3_ /PP composite exhibited larger normalized swell ratio B(τ,ϕ)/B(τ,ϕ=0) at low shear stress while smaller normalized swell ratio at high shear stress than modified Ca_2_CO_3_ /PP composite.

f(ϕ) is summarized in Table 3. It is found that f(ϕ)=b−lτ can reflect one linear correlation. However, b and l become larger with higher filler content as illustrated in Figure 4. b is the linear growth function of filler volume percentage while l is exponential function of filler volume percentage. The latter case is also well predicted by Equation 9.

**Table 3:** f(ϕ) for iPP/ Ca2CO_3_ nanoparticles of various filler loadings.

**Filler content (%)**	f(ϕ) **for iPP** **/stearic acid-coated** **Ca_2_CO_3_**	f(ϕ) **for iPP/ uncoated CaCO_3_**f(ϕ)=b−lτ	
		b	l
5%	0.98883	0.9975	2.578E-7
10%	0.9637	0.9965	7.698E-7
15%	0.9602–4.366x10^-7^ τ	0.9948	7.688E-7
20%	0.9113	1.0115	1.609E-6
25%	0.9164–8.1x10^-7^ τ	1.1120	4.340E-6
Fitted equations	f(ϕ)=1 - ϕ/255.4	b = 0.94926(1+ ϕ/195)	$\text{l = 0.0085}\phi^{2.616}$

#### 3.2.3. Application of Swell Model to Fluoroelastomer Nanocomposites

**Figure 5:**
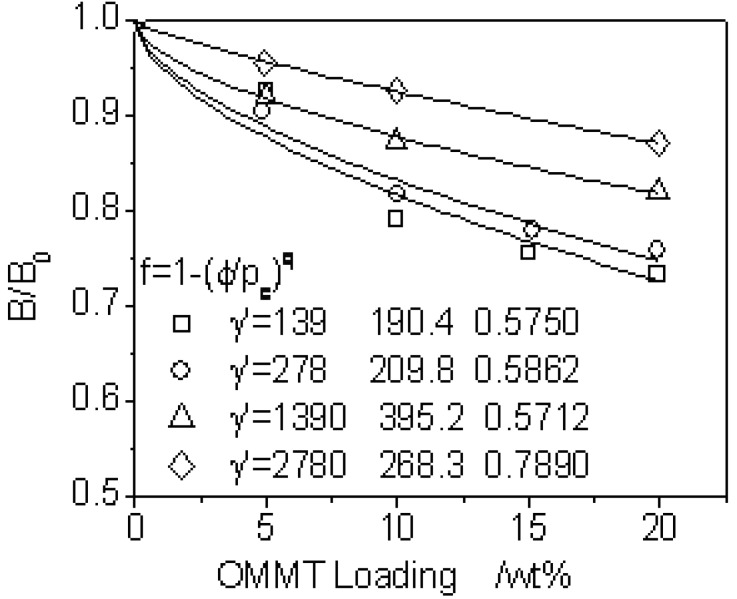
Relative die-swell as a function of OMMT content at four different shear rates.

B(τ,ϕ)/B(τ,ϕ=0) against ϕ can be obtaned by Equation (8) as illustrated in Figure 5 for OMMT filled fluoroelastomer nanocomposites [36]. The better regulation may also be correlated for long L/D = 30:1 and entrance angles of 45°and 60°. At one level of shear rate, Equation (9) is well defined with certain $p_{c}$ and q, which together grow when shearing the melt more strongly. Addition of the inorganic particles into polymer matrix prevents the chains from orientating to a higher degree at the entry region resulting in lower die swell out of the capillary exit. This effect becomes more significant for higher loaded composites. On the other hand, this effect becomes weaker at higher shear rate since the composite structure is, after all, of polymer matrix whose chains is apt to recoil and re-entangle after storing more energy in being elongated or sheared. *i.e.*, the extrudate swell becomes greater at higher shear rate, as illustrated in Figure 5.

#### 3.2.4. Application of Swell Model to Quasi-Nanogel Particle-Filled Natural Rubber

**Figure 6:**
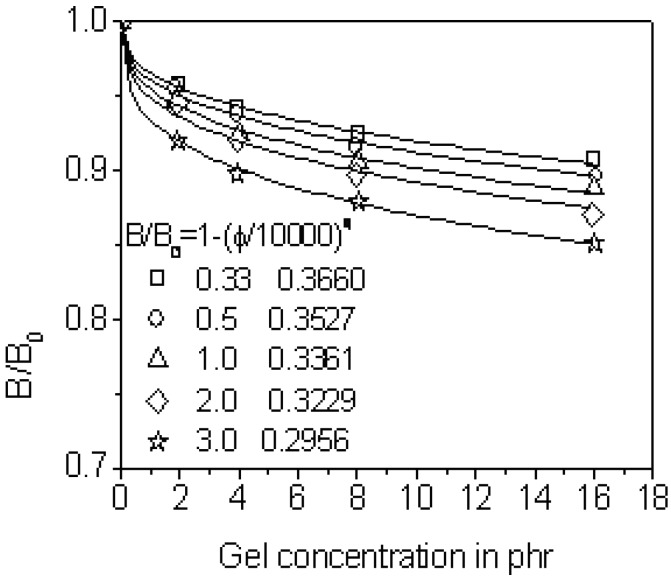
Effects of the sulfur-crosslinked gel loading on the die swell property of raw NR.

Ref. [29] reported that gel content and its crosslink density becomes greater with the sulfur-to-accelerator ratio. The sulfur-to-accelerator ratio controls the vulcanization dynamics and subsequently the gel size/content and the crosslink density. The resulting natural rubber exhibited higher tensile strength against lower elongation at break. For the rubber with different degrees of crosslinked gels [37], the extrudate swell through one long capillary of a length-to-diameter ratio equal to 30 can also be explained using Equation 8 as shown in Figure 6. The fitted $p_{c}$ remains nearly invariable while q decreases. This illustrates that q reflects the stability of filler network relative to the rubber crosslink structure and their harmony in flow. The pseudocrosslinked gel filler acted as one ‘spring’ in series with vulcanized rubber ‘spring’. Elongated filler ‘spring’ retracted to absorb some elastic energy stored by rubber matrix ‘spring’ to swell resulting in smaller die swell. In a certain sense, this seems to prove the formation of double network-structure in nanocomposites as in the present case.

**Figure 7:**
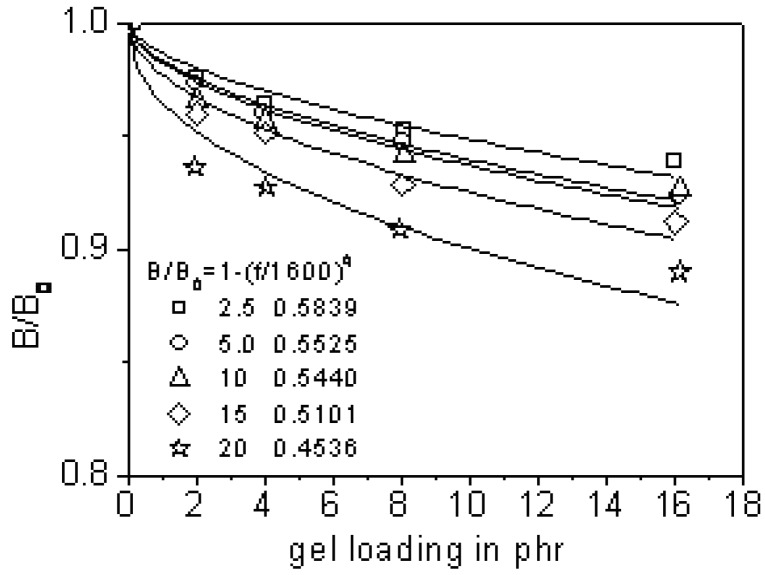
Variation in the relative die swell with gel concentration for electron beam-crosslinked NR latex gel-filled NR systems at 130 °C and 122.5 s^-1^.

The inclusion process of filler into the matrix probably results in different composite microstructures and subsequently a varied swell behavior. Different from the sulfur-vulcanization, discussed in following section is the swelling of EB-radiated quasi-nanogel particles filled natural rubber [37]. It is reported in [37] that the filler gel content and crosslink density became higher while gel size became smaller when radiated at higher dose. Die swell of natural rubber was weakened to a greater degree after being filled with more radiated gel at the same shear rate, as shown in Figure 7. At the same filling level, swell ratio dropped when the filler gel was cross-linked to a larger density and smaller sizes. This again illustrated the existence of filler pseudo-network, rubber entangled network and filler-rubber mesoscopic network. The interlocked networks restricted the swelling in extrusion.

**Figure 8:**
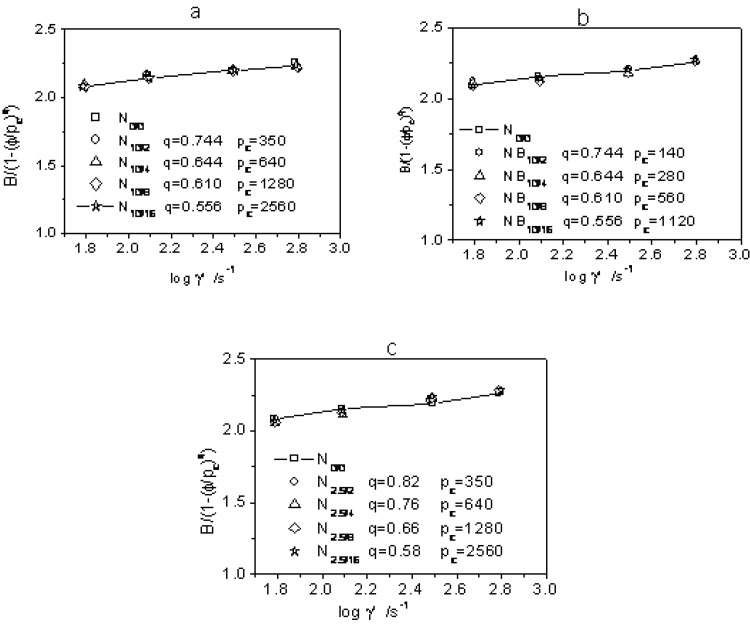
Die swell *vs.* apparent shear rate plots for EB-cross-linked NR latex gel-filled raw NR for gels irradiated: (a) without a sensitizer at 10kGy; (b) without a sensitizer at 2.5kGy; and (c) with a butyl acrylate sensitizer at 10kGy, respectively.

Figure 8 shows the effects of filler concentration on the $B(\dot{\gamma },\phi )/f(\phi )$ *versus* shear rate. Plots a-c all confirmed the rationality of Equation 8. The swell ratio increases with shear rate, which reflects the origin of the swell is elastic recovery. For the same filler, $p_{c}$ increases while q decreases when filler concentration is increased.

Comparison of Figure 8a with 8b shows that the butyl acrylate sensitizer-induced crosslinked gel filler reduced the rubber swell to a higher degree than the no sensitizer-induced one at the same radiation dose and filler concentration. After sensitizer-induction, $p_{c}$ reduces while q keeps almost constant. It is also reported in [37] that the filler gel content and crosslink density became higher while gel size turned smaller when sensitizer was used at the same radiation dose. Smaller and closer sensitizer-induced crosslinked gel more easily become fragile pile-ups compared to the non-sensitized one. Thus, the percolation becomes lower as being representative by smaller $p_{c}$.

At the same gel filler concentration, the weaker radiation corresponds to lower gel content and smaller crosslink density in filler particles while gel size turned greater. Comparison of Figure 8a with 8c shows that q reduces while $p_{c}$ remains almost constant for samples with filler more strongly radiated. Under strong radiation, gel chains were crosslinked to a higher degree, which interact with less rubber matrix at the interface such that smaller q illustrates the less dependent of swell ratio with the filler-content.

## 4. Conclusions

A unified extrudate swell correlation with material properties and capillary parameters was developed for polymer melts and their nanocomposites in long and short capillary extrusion when considering reservoir entry effect derived from Song’s molecular dynamics. Compared to the concentration shift factor in dynamic moduli for composites, the fact that die swell decrement with filler content can well be described by a similar concentration shift factor to that of the pure polymer matrix was pointed out for the first time. The factor is a function of the shear field (stress or shear rate), filler content, filler internal structure and the surface state as well as the matrix properties. The quantitative model fit well for five kinds of nanocomposites. The excellent agreement of model-predicted value with the experimental data verified the certain rationality for the proposed composite swell theory. To a certain degree, this showed that swell decrement with filler content is due to the complex variations in material microstructure.
